# When genes turn traitor: *de novo* transcriptomics uncovers pearl millet’s rancidity machinery

**DOI:** 10.3389/fpls.2025.1677082

**Published:** 2025-11-17

**Authors:** Ranjeet R. Kumar, Suneha Goswami, Vinutha Thimmegowda, Nitin Singhal, Ulaganathan Mabalirajan, Gyan P. Mishra, Girish K. Jha, Gyanendra K. Rai, Shelly Praveen, Chellapilla Tara Satyavathi

**Affiliations:** 1 Division of Biochemistry, Indian Council of Agricultural Research (ICAR)-Indian Agricultural Research Institute, New Delhi, India; 2 Department of Biotechnology (DBT)-National Agri-Biotechnology Institute, Mohali, Punjab, India; 3 Council of Scientific and Industrial Research (CSIR)-Indian Institute of Chemical Biology, Kolkata, India; 4 Division of Seed Science and Technology, Indian Council of Agricultural Research (ICAR)-Indian Agricultural Research Institute, New Delhi, India; 5 CABin, Indian Council of Agricultural Research (ICAR)-Indian Agricultural Statistical Research Institute, New Delhi, India; 6 Sher-E-Kashmir University of Agriculture Science and Technology, Jammu, India; 7 Indian Council of Agricultural Research (ICAR)-Indian Institute of Millet Research, Telangana/Andhra Pradesh, Hyderabad, India

**Keywords:** rancidity, DEGs, differentially expressed transcripts, lipase, landrace, lipids

## Abstract

Pearl millet flour is highly nutritious but prone to rancidity, leading to off-odor development and reduced shelf life. To understand the molecular basis of this phenomenon, we performed *de novo* transcriptome sequencing on diverse pearl millet genotypes (landraces, hybrids, and composites) and identified 219,965 genes and 386,184 transcripts with functional annotation revealing key pathways linked to lipid and starch degradation. Differential gene expression (DGE) analysis identified significant upregulation of rancidity-linked genes [lipases (LIPs), lipoxygenases (LOXs), peroxidases (POXs), and polyphenol oxidases (PPOs)] in high-rancid genotypes. Data mining for characterizing rancid pathway showed the presence of 2,038 *LIP*, 209 *Lox*, 26 hydroperoxide lyase (*HPL*), 1,023 *POX*, and 17 *PPO* genes. Tissue-specific expression analysis of variants of *Lip*, *Lox*, *Pox*, and *PPO* during the different sub-stages of endosperm development showed an abundance of transcripts of *Lip-2*, *LOX-3*, and *POX-4* during the seed hardening stage. Enzymatic assays confirmed increased LIP (up to 200.5 µmol h^-1^ g^-1^), LOX (184 nM HPOD min^-1^ mg^-1^), POX, and PPO activities in stored flour, correlating with rancidity progression. Notably, landraces exhibited lower expression of rancidity-linked genes compared to hybrids and composites, suggesting genetic variability in flour shelf life stability. Our study provides the first comprehensive transcriptomic resource for pearl millet rancidity, identifying candidate genes and enzymatic markers for future breeding programs aimed at improving flour storage quality.

## Introduction

Pearl millet is considered as “God’s grain” because it has all the beneficial nutrients required in our balanced diet (Kumar et al., 2022b). This pseudo-grain crop was very much popular among our ancestors, but with the domination of cereals, it was slowly depleted from our plate (Satyavathi et al., 2021). In the post-green revolution era, millet fields have been replaced with fields that grow other cereal crops like wheat, rice, and maize. Food consumption pattern and the lack of diversity in food items lead to malnutrition in most parts of the world (Srivastava et al., 2022). The nutrient content of grains is depleting day-by-day due to adverse environmental conditions (Ashoka et al., 2023).

Pearl millet is a hardy crop and has inherent potential to survive under adverse environmental conditions with limited consumption of natural resources. The pearl millet grains are rich in carbohydrate (~65%–70%), lipid (~6%–7%), protein (10%–12%), fiber (~3%–3.5%), and micronutrients like Fe [~70 parts per million (ppm)] and Zn (~40 ppm) (Goswami et al., 2020). The short shelf life of the flour is one of the major hurdles in the production and utilization of pearl millet. Among all the millets and cereals, the pearl millet grain has maximum lipid content (~6%–7%) localized in the outer cover of the grains (Kumar et al., 2022a). The grains have very well-defined compartments for catabolic enzymes like α/β-amylases (Amy), lipases (Lip), proteases, lipoxygenase (LOX), peroxidase (POX), and polyphenol oxidase (PPO). These enzymes are localized in the germ tissue and are separated from their respective substrates dispersed in the endospermic tissue (Kumar et al., 2021; Aher et al., 2022). The micronutrients, especially iron and zinc, are localized in the outer cover of the grains (Srivastava et al., 2022). Recently, it has been reported that the quality of the pearl millet grains also deteriorates after long storage. Off-odor development, bitterness, and rancidity are exaggerated with the milling of grains into flour (Goswami et al., 2020). Upon milling, the catalytic enzymes localized in the germ tissue are exposed to their substrate like starch (α/β-amylases), lipid (lipase), and protein (proteases). Because of the high lipid content (~6%–7%), the lipase acts on these lipids and form free fatty acids (FFAs) along with glycerol (Kumar et al., 2021). FFAs are essential in our diet for various metabolic processes. FFAs serve as a substrate for the LOX enzyme, which oxidizes it into hydroperoxides. These hydroperoxides are further acted upon by hydroperoxide lyase (HPL) and POX to form various carbonyl compounds like aldehyde and alcohol. These carbonyl compounds are mainly responsible for the off-odor and bitterness of the flour. Even the browning of the flour happens due to the sequential reaction of PPO on polyphenols present in the flour (Ali et al., 2023a). The pearl millet flour has a very short shelf life [7–12 days after milling (DAM)], which restricts its use in food processing and manufacturing industries.

The advancement in basic tools and techniques has facilitated the characterization of various biological pathways in agriculturally important crops. It led to the discovery of key genes/proteins/metabolites associated with different processes within the plant system. The genes/enzymes responsible for the off-odor development and bitterness in pearl millet flour have not yet been elucidated. Few variants of rancidity-causing genes have been partially characterized in crops like rice, oats, soybean, and rye (Alomari et al., 2018).

Next-generation sequencing (NGS) has revolutionized the world of research and has provided immense opportunity to identify novel genes/proteins (Satam et al., 2023). Different omics tools have been used recently in crops to identify novel genes, hypothetical proteins, metabolites, and their involvement in different pathways (Gondane and Itkonen, 2023, Sinha et al. 2011). RNA sequencing (RNA-seq) is a method of characterizing or identifying the whole set of mRNA or genes present in a sample (Fan et al., 2013). It provides ample opportunity to identify the potential genes and understand their function and regulation. It also helps in mapping the pathway and understanding the mechanism of any biological process operating inside the system (Wang et al., 2009).

Here, our primary objective is to identify candidate genes responsible for encoding proteins/enzymes that contribute to the off-odor development and rancidity in pearl millet flour. We aimed to dissect the molecular mechanism underlying off-odor development and rancidity by correlating genes with enzymes, lipid degradation, and associated oxidative processes.

## Materials and methods

### Samples and growth conditions

We have selected two landraces (Chadhi Bajri and Damodhar Bajri), one hybrid (Pusa-1201), and one composite (Pusa-701) of pearl millet for the present investigation. The seeds were collected from different agro-climatic zones in India with the help of ICAR-All India Coordinated Research Project (AICRP) on pearl millet, Jodhpur, India. The seeds were first pre-treated with Bavistin at 0.25% and plants were grown in rectangular plastic pots of dimensions 66 × 21 × 15cm (L × W × H) inside the Nanaji Deshmukh Plant Phenomics Centre at ICAR-Indian Agricultural Research Institute, New Delhi, India. Equal quantities of farmyard manure (FYM) and sand were used for filling the pots, and standard procedures were followed for the inter-cultural operations (Kumar et al., 2020b). The experiment was carried out with three biological replicates. The plants were grown under 28 ± 2°C during vegetative/pollination and 32 ± 2°C during spike development/grain-filling with a 12-h light/dark cycle and a relative humidity of ~75% during different stages of growth and development. The samples (leaf, stem, and spike) for the *de novo* transcriptome sequencing were collected during the grain-filling (sub-stage seed hardening). Samples for the expression analysis were collected at different developmental stages. The collected samples were immediately frozen in liquid N_2_ and stored in a −80°C deep freezer for further downstream application.

### *De novo* transcriptome sequencing

#### Total RNA isolation and library construction

The total RNA was isolated from different tissues (leaf, stem, and spikes) of Chadhi Bajri, Damodhar Bajri (landraces), Pusa-1201 (hybrid), and PC-701 (composite) using the Trizol method (Chomczynski, 1993). The purpose of using different tissues was to understand the gene network and expression dynamics operating in developing grains. The quality and integrity of the isolated RNA were checked using NanoDrop One (Thermo Fisher Scientific) and by resolving the sample on 1.2% agarose gel. The total RNA with two distinct bands and an OD_260_/OD_280_ ratio of ~2.0 was further used for pooling and library construction. Pooled RNA sample (~400 ng) was used to prepare RNA-seq libraries using the Illumina Truseq Stranded mRNA sample preparation kit (Illumina Inc., San Diego, CA, USA). The pooled samples were subjected to paired-end sequencing (2× 150 bp) on the Illumina HiSeq™ 2500 system (Illumina Inc., San Diego, CA, USA) following the protocols as mentioned in our earlier publication (Kumar et al., 2015).

### *De novo* assembly and gene annotation

The quality of raw reads generated was checked using FASTQC v 0.11.9 (https://bioweb.pasteur.fr/; Andrews, 2010). We have used AdapterRemoval v2.3.3 (https://github.com/MikkelSchubert/adapterremoval/releases/tag/v2.3.3) to remove the adapter sequences and low-quality bases. We kept the minimum read length to 30 with a Phred Score of 20 in the trimming step. The raw data were aligned with the Silva database using Bowtie2 v 2.3.5.1 for rRNA removal. All trimmed paired reads data were used to assemble the transcriptome using Trinity v 2.15.1 with default parameters (https://github.com/trinityrnaseq/trinityrnaseq/releases/tag/Trinity-v2.15.1). We have further used the TrinityStats pearl script to examine the statistics of the assembly. The expression of the differentially expressed genes (DEGs) was carried out using Kallisto v 0.46.1 (https://github.com/pachterlab/kallisto) with default parameters. All assembled transcripts were subjected to annotation using the Rice Database [ftp://ftp.ensemblgenomes.org/pub/plants/release-46/fasta/oryza_sativa/pep/Oryza_sativa.IRGSP-1.0.pep.all.fa.gz] and NCBI ANNOTATIONX v 2.6.0+, and the search was performed against UniProt databases (https://www.uniprot.org/) based on homology. The annotation was carried out based on best hit and in-house pipeline. The differential gene expression (DGE) analysis was carried out using edgeR software v 3.19 (https://bioconductor.org/) with default parameters, and the results were filtered based on a log_2_ fold change of +2/−2 and *p*-value ≤ 0.05.

### Validation of RNA-seq data using quantitative real-time PCR

To validate RNA-seq data, pooled samples from hybrid (Pusa-1201) collected during the grain-filling stage were used for the total RNA isolation, cDNA synthesis, and quantitative real-time PCR (qRT-PCR) (Kumar et al., 2020a). We have randomly selected 10 differentially expressed transcripts (DETs) from the RNA-seq data and designed transcript-specific primers to be used for the qRT-PCR (**Supplementary Table S1**). The DETs selected for the expression analysis were Serine/threonine kinase (*STK*; transcript no. DN2506), Lipase class-III (*Lip-III*; transcript no. DN63949), Heat shock protein 70 (*HSP70*; transcript no. DN1582), Glucoside hydrolase (*GH*; transcript no. DN15277), UDP glucosyltransferase (*UGT*; transcript no. DN51146), Phenyalanine lyase (*PAL*; transcript no. DN8260), Sucrose transporter (*SUT*; transcript no. DN48102), Glutamate dehydrogenase (*GDH*; transcript no. DN254), Mn-superoxide dismutase (*MnSOD*; transcript no. DN131007), and Lipoxygenase (*LOX*; transcript no. DN7868). The oligos were synthesized commercially and used for the qRT-PCR expression analysis following the steps mentioned in our earlier publication (Kumar et al., 2020a). The relative quantification of mRNA was performed on the CFX96 platform (Bio-Rad, UK) using three biological replicates. Actin-7 gene (accession no. KM105957.1) was used as an endogenous control gene for normalizing the *C*_t_ value and the fold expression was calculated using the Pfaffl method (Pfaffl, 2001). The fold expression (2^−ddCt^ value) was converted to the absolute value of log_2_ fold change for comparative analysis with the RNA-seq dataset and for establishing the correlation between the RNA-seq and qPCR datasets.

### Identification of genes associated with pathway responsible for flour rancidity

We have retrieved the coding sequences of some of the genes linked to rancidity, such as *Lip*, *LOX*, *POX*, and *PPO* from RAP-DB (Rice Annotation Project Database) and The Arabidopsis Information Resource database (TAIR, www.arabidopsis.org). The sequences were used as queries to search their orthologs in our transcriptome assembled sequences.

### Expression analysis of rancidity-causing genes in endosperm and harvested grains

The potential transcripts showing homology with enzymes responsible for off-odor development in flour were identified, and furthermore, a few of them were selected (based on digital fold expression) for their expression analysis at different sub-stages of endosperm development (S_0_, button stage; S_1_, milky-ripe; S_2_, mealy-ripe; S_3_, seed hardening; S_4_, harvested grains) in pearl millet cv. Pusa 1201. The genes selected for the expression analysis were lipase [*Lip-1* (accession no. OQ184871), *Lip-2* (accession no. MZ590565), *Lip-3* (accession no. MZ590564), and *Lip-4* (accession no. OQ304605)], lipoxygenase [*LOX-1* (accession no. OQ184873), *LOX-2* (accession no. OQ184874), *LOX-3* (TRINITY_DN10043_c0_g1_i1), and *LOX-4* (TRINITY_DN118950_c0_g1)], peroxidase [*POX-1* (TRINITY_DN109983), *POX-2* (TRINITY_DN177283), *POX-3* (accession no. PP171489) & *POX-4* (TRINITY_DN115862)], and polyphenol oxidase [*PPO-1* (accession no. PP765147), *PPO-2* (TRINITY_DN183701_c0_g1), *PPO-3* (TRINITY_DN167165_c0_g1), and *PPO-4* (TRINITY_DN112679_c0_g1)]. The transcript-specific primers were designed using the Genefisher2.0 primer designing software (https://bibiserv.cebitec.uni-bielefeld.de/genefisher2) and were synthesized commercially (**Supplementary Table S1**). The tissues (different sub-stages of endosperm development) along with flour were used for the total RNA isolation using the RaFlex Total RNA Isolation Kit (Geneilabs). Other steps were followed as mentioned in our earlier publication (Kumar et al., 2020a). Actin-7 gene (accession no. KM105957.1) was used for normalizing the *C*_t_ value. The relative fold expression was calculated using the Pfaffl method (Pfaffl et al., 2002).

### Activity assay of rancidity-causing enzymes in developing endosperm and flour

Based on our findings in the present investigation and the information retrieved from other literature, we performed activities of enzymes linked to off-odor development/rancidity like LIP, LOX, POX, and PPO in flour 0 (fresh flour, M_0_), 10 (M_1_), 30 (M_2_), 60 (M_3_), and 90 (M_4_) DAM.

#### Preparation of enzyme extract

We have optimized the extraction conditions (pH, molarity, and buffer) through a pilot experiment, and furthermore, 1g of flour was used for the enzyme extract preparation by homogenizing it in 10 mL of 0.2 M K_2_HPO_4_ buffer (pH 7.5). The homogenate was centrifuged at 10,000 rpm for 20min in a refrigerated centrifuge. The supernatant collected was filtered through four layers of cheese cloth and was used as enzyme extract for the activity assay. We have used the Bradford method for the estimation of total soluble protein (Bradford, 1976).

### Activity assay of lipase and lipoxygenase enzymes

The activity assay of LIP was performed following the method of Itaya and Ui with some modifications (Itaya and Ui, 1966). A reaction mixture was prepared by adding 1 mL of substrate [0.98% (w/v) NaCl, 200 µL of Tween 20, and a mixture of olive oil and water in a 1:3 ratio] and 100 µL of enzyme extract and was incubated at 37°C for 15min; other steps were followed, as mentioned in the protocol. The LIP activity was calculated using the standard curve of oleic acid and expressed as nanomolar FFAs min^−1^ mg^−1^ soluble protein. The activity assay of the LOX enzyme was carried out as mentioned in our earlier publication (Goswami et al., 2020). The molar extinction coefficient of linoleic acid (25 mM^−1^ cm^−1^) was used for calculating the activity of LOX, and the result was presented as nanomolar hydroperoxide min^−1^ mg^−1^ soluble protein (nM HPOD min^−1^ mg^−1^ soluble protein).

### Activity assay of polyphenol oxidase and peroxidase enzymes

We have assayed the PPO activity following the protocol of Almeida et al. (2019). In brief, a reaction mixture was prepared by adding 50 μL of enzyme extract along with 1,450 μL of phosphate buffer (0.2 M, pH 6.0) and 1,500 μL of catechol (0.2 M). The absorbance of RM was taken at 425 nm for 1, 2, and 3 min with 30-s intervals between the readings. The activity of PPO was calculated using the molar extinction coefficient of catechol (3,400 mM^–1^ cm^–1^) and was expressed as μmol min^–1^ g FW^–1^.

Similarly, the POX activity assay was performed following the method mentioned above (Almeida et al., 2019). In brief, a reaction mixture was prepared by adding 50 μL of enzyme extract along with 1,750 μL of phosphate buffer (0.2 M, pH 6.0), 100 μL of guaiacol (5 g L^–1^), and 100 μL of hydrogen peroxide (0.8g L^–1^). The absorbance was taken at 470 nm for every 1, 2, and 3 min. The activity of POD was calculated using the molar extinction coefficient of guaiacol (26.6 mM^–1^ cm^–1^) and expressed as μmol min^–1^ g FW^–1^.

### Statistical analysis

We have used three biological replicates for the biochemical and molecular analysis. The data generated were characterized using one-way analysis of variance (ANOVA) conducted using SPSS (SPSS Inc., USA) (*p* ≤ 0.05).

## Results and discussion

Pearl millet, often classified as an orphan crop, remains underexplored at the molecular level, particularly with respect to genes and proteins associated with key biological processes. One of the major challenges limiting its wider adoption is the rapid rancidity and off-odor development in its flour after milling (Ali et al., 2023b). However, there is a significant knowledge gap regarding the genetic, molecular, and proteomic basis of this phenomenon. The biochemical pathway responsible for the rancidity, along with its key molecular components, remains largely uncharacterized in pearl millet.

### *De novo* transcriptome sequencing of pooled samples (leaf, stem, and spikes)

Here, we performed *de novo* transcriptome sequencing of pooled samples from diverse cultivars of pearl millet to identify candidate genes and their potential functional roles in flour rancidity. Total RNA was isolated from leaves, stem, and spike tissues during the seed hardening stage and used for library construction, following standard approaches reported for cereal transcriptomics (Varshney et al., 2017). The RNA-seq was performed using the Illumina Hiseq-4000 platform. We have generated ~8–10 Gb raw data per sample with GC% in the range of 46–49 and a Q30 value of 97 (**Table 1**). Base quality score distribution showed an average base quality Q30 of 97.32% (error probability ≥ 0.001). The findings were consistent with the high-quality sequencing benchmarks (Kulkarni et al., 2016). The high-quality reads from all the 12 samples were used to generate one pooled assembly using the Trinity assembler with default options, as successfully applied in several cereal *de novo* assemblies (Grabherr et al., 2011). The summary of transcriptome assembly results is presented in **Supplementary Table S2**. The length distribution for all assembled transcripts is presented in **Supplementary Figure S1**. Using the Trinity assembler, we have identified 219,965 genes and 386,184 transcripts in diverse genotypes of pearl millet with a ~GC of 47.98%. We have observed the average contig length of 1,208.13 with an N50 value of 2,614. The total number of assembled bases was 154,468,863 with a maximum average transcript length of 200–250 bp. RNA-seq has been used in the past to understand the regulation of hormones, seedling germination, and mechanism of abiotic and biotic stress tolerance in pearl millet (Wu, 1995; Shivhare et al., 2020).

**Table 1:** Raw data summary generated from *de novo* transcriptome sequencing of pooled samples of landraces (Chadhi Bajri and Damodhar Bajri), Hybrid (Pusa-1201), and composite (PC-701) of pearl millet.

Sample name	Number of reads	Number of bases (Mb)	GC (%)	Q30
CHADI-BAJRI-POOL	9,08,18,954	9,081.9	49.16	95.595
DAMODHAR BAJRI-POOL	14,34,54,980	14,345.5	49.65	97.575
PC701-POOL	10,25,67,450	10,256.74	45.975	97.55
PUSA-1201-POOL	10,24,62,724	10,246.28	48.095	97.675

### Annotation of transcripts generated using *de novo* transcriptome sequencing

All assembled transcripts were subjected to annotation using the Rice Database and NCBI ANNOTATIONX v 2.6.0+. We observed 42,355 coding sequences by mapping on the Rice Database. We observed 370,201 sequences (95.86%) based on homology using Top-hit (**Supplementary Table S3**). Furthermore, characterization for the identification of genes based on the Rice Database showed the presence of 370,201 genes (**Supplementary Table S4**). We have observed 34,890 rice coding sequences showing homology with Trinity sequences. The number of trinity sequences that remains unannotated was ~15,983.

### Differential gene expression analysis

We have identified the DGEs by comparing the hybrid and composite with landraces (considered as the hidden treasure of genes). DGE was performed using the edgeR program; we used 386,184 transcripts for the analysis, and DEGs were filtered at log_2_ fold change with a cutoff of +2/−2 (*p*-value of ≤0.05) (**Supplementary Table S5**).

**Figure 1:**
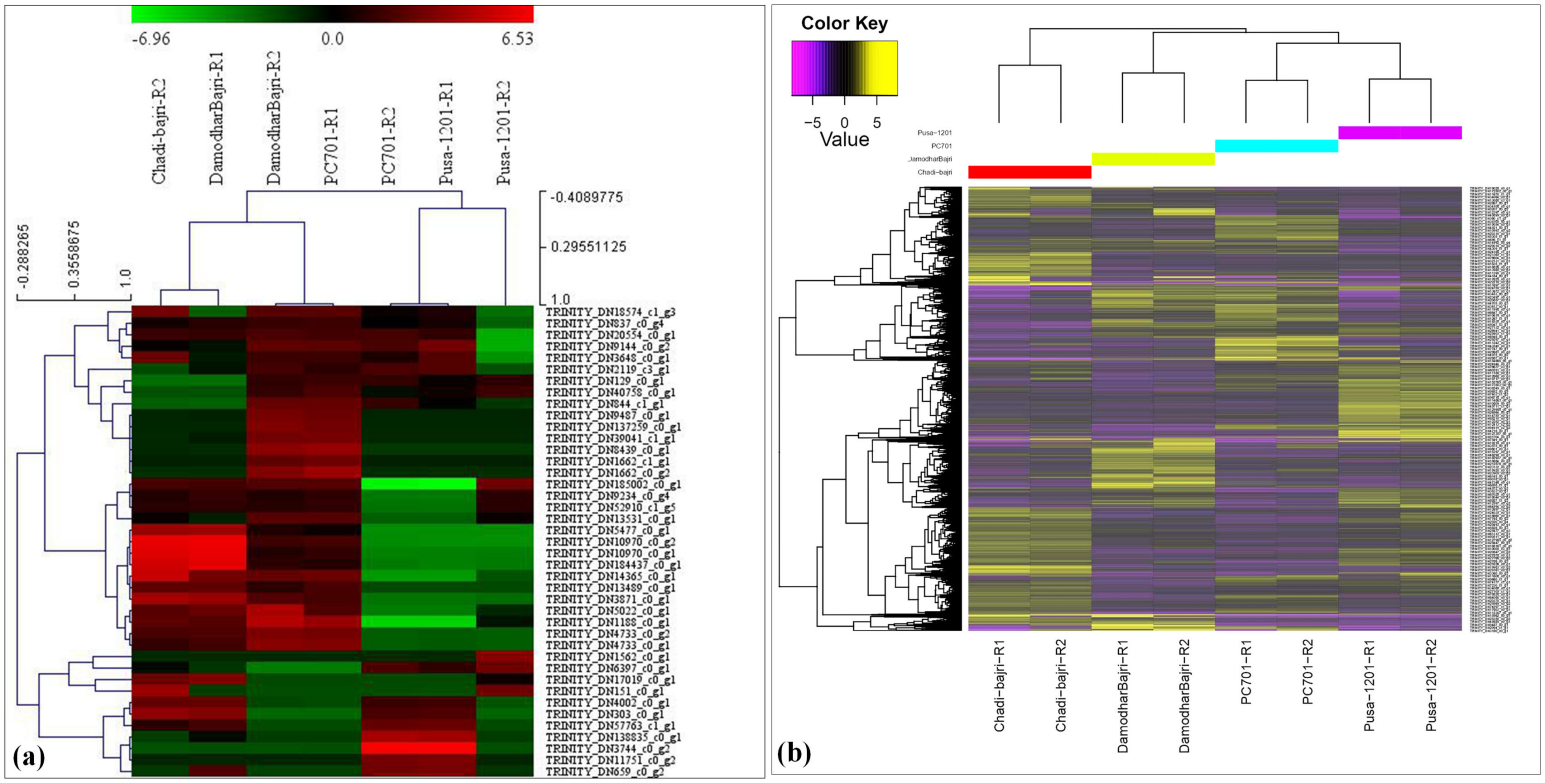
Differential gene expression (DGE) profiles in pearl millet genotypes. **(a)** Heatmap of the top 10 differentially expressed (DE) genes for each pairwise comparison (log_2_ FC ≥ 2, FDR ≤ 0.05). **(b)** Hierarchical clustering of all significant DEGs across landraces (Chadhi Bajri and Damodhar Bajri), hybrid (Pusa 1201), and composite (PC-701) genotypes. Columns indicate biological replicates; rows represent individual genes. Color scale: yellow/red = upregulation (log_2_ FC > 0), dark violet/green = downregulation (log_2_ FC < 0), dendrograms reflect Euclidean distance-based clustering of samples (columns) and genes (rows).

Comparative analysis among the landraces (Damodhar Bajri and Chadhi Bajri) showed the presence of 2,834 downregulated and 2,158 upregulated genes (**Supplementary Table S6**). We observed very significant differences in the expression of up- and downregulated genes (DEGs) among the landraces, with Chadhi Bajri having more DEGs, as compared to Damodhar Bajri. A similar pattern of expression was observed, when we compared the presence of DEGs in PC-701 (composite), as compared to Chadhi Bajri (landrace). The DEGs were observed to be maximum in landraces, as compared to composites. Comparative analysis of Pusa-1201 (hybrid) with Chadhi Bajri (landrace) showed maximum DEGs (downregulated—2,623) in hybrid, as compared to landrace. Similarly, comparative analysis of Pusa-1201 and Pusa-701 showed 2,186 upregulated and 2,077 downregulated genes. Stage-specific RNA-seq analysis showed the presence of 155 upregulated and 251 downregulated (during flowering stage) and 349 upregulated and 378 downregulated transcripts (during milking stage) linked to the regulation of Fe and Zn pathways (Satyavathi et al., 2021). We have also plotted a heatmap of the top 10 differentially expressed (DE) features within each pairwise comparison and observed maximum upregulated transcripts in PC-701 and downregulated transcripts in Pusa-1201 (**Figure 1a**). The expression of the top 10 DE showed significant variations among the landraces, hybrids, and composites. We also generated a heatmap of DEGs identified in the present investigation (**Figure 1b**). We observed the abundance of upregulated transcripts in Pusa-1201 and PC-701, whereas the downregulated transcripts were observed to be more abundant in landraces.

We have also generated a volcano plot in order to understand the pattern of DEGs among the landraces, hybrids, and composites. Volcano plot analysis of Damodhar Bajri with Chadhi Bajri showed a very discrete and concentrated appearance of DEGs (**Supplementary Figure S2a**). Similarly, volcano plot analysis of Pusa-1201 with Chadhi Bajri showed a very scattered pattern of up- and downregulated transcripts in hybrids, as compared to landraces (**Supplementary Figure S2b**). Comparative analysis of DEGs in PC-701 and Damodhar Bajri showed partially dispersed distribution of transcripts with the majority showing non-significant differences in expression (**Supplementary Figure S2c**). Volcano plot analysis of DEGs in Pusa-1201 with PC-701 showed a very wide distribution of downregulated transcripts, as compared to upregulated transcripts (**Supplementary Figure S2d**). Most of the DEGs (up- and downregulated) were observed to overlap in Pusa-1201 and PC-701 of pearl millet.

Comparison of the top 10 DE based on genes showed a much higher expression of genes in landraces compared with composites and hybrids, as evident from the color code. The findings are in conformity with the observation of Varshney et al. (2017). A *de novo* transcriptomic approach has been used to identify 1,396 up- and 936 downregulated and 1,000 up- and 1,591 downregulated transcripts in resistant inoculated/resistant control and susceptible inoculated/susceptible control of pearl millet (Kulkarni et al., 2016).

### Gene Ontology analysis of annotated transcripts

**Figure 2:**
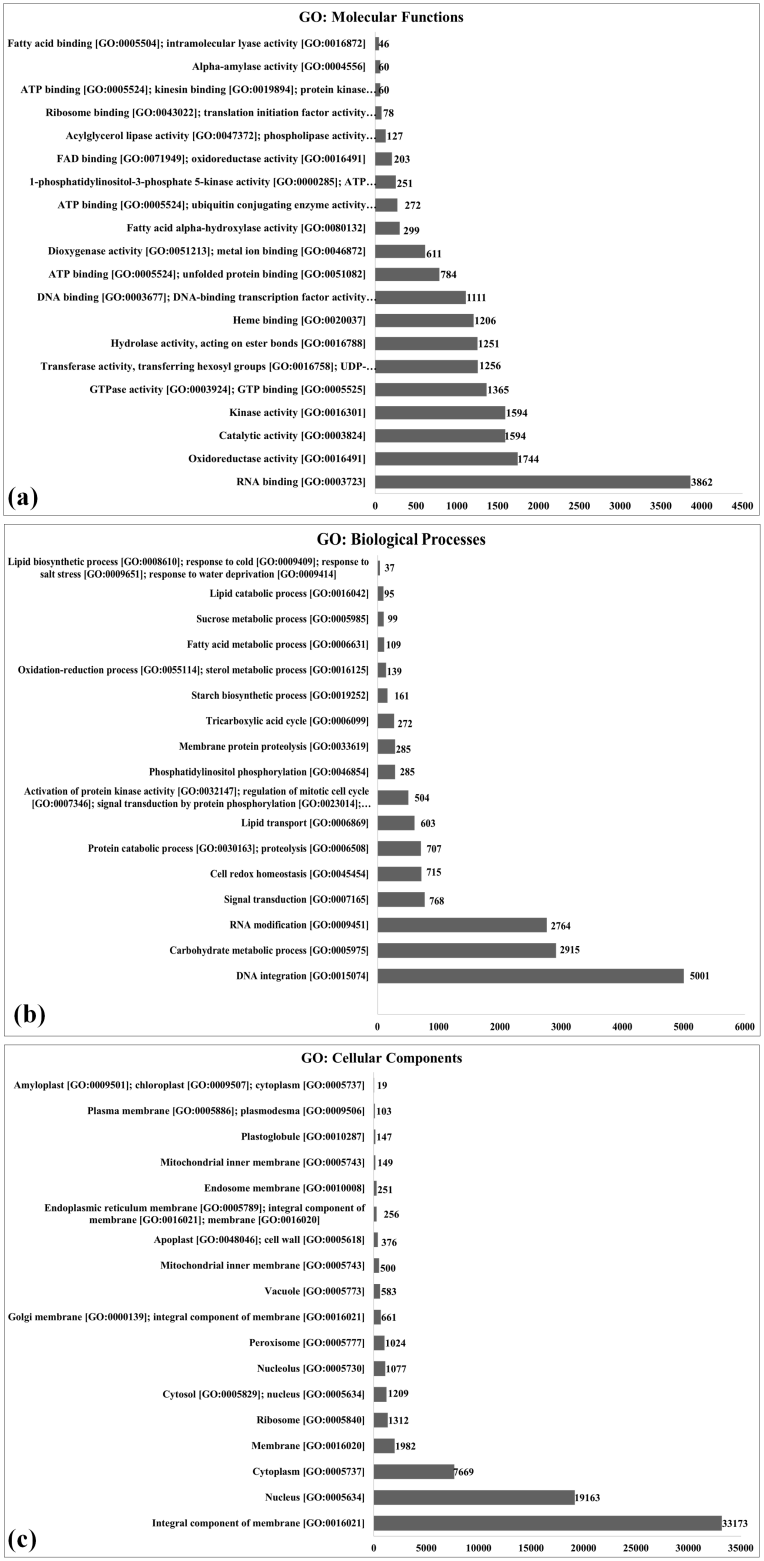
Gene Ontology (GO) enrichment analysis of differentially expressed genes (DEGs) in pearl millet genotypes, Functional categorization of DEGs identified through *de novo* transcriptome sequencing is shown for **(a)** molecular function, **(b)** biological process, and **(c)** cellular component. The *X*-axis indicates the number of DEGs assigned to each GO term (count), while the *Y*-axis lists the significantly enriched GO terms (*p* ≤ 0.05); DEGs were derived from comparisons between landraces (Chadhi Bajri and Damodhar Bajri), hybrid (Pusa 1201), and composite (PC-701) genotypes.

The 370,201 transcripts identified were further subjected to functional annotation using the Gene Ontology (GO) analysis and were classified into three groups: GO (molecular function), GO (biological process), and GO (cellular components) (**Figure 2**; **Supplementary Table S7**). GO analysis of annotated transcripts based on molecular functions showed 3,862 transcripts to be involved in RNA binding [GO:0003723], followed by 1,744 transcripts in oxidoreductase activity [GO:0016491]. Very few transcripts were observed to be involved in fatty acid binding [GO:0005504]. GO analysis revealed the flavonoid pathway, lignin biosynthesis, and phenyl propanoid pathway to be more enriched in pearl millet during the flowering stage (Shivhare et al., 2020).

Similarly, GO analysis of annotated transcripts based on biological processes showed a maximum of 5,001 transcripts to be involved in DNA integration [GO:0015074] followed by 2,915 transcripts involved in the carbohydrate metabolic process [GO:0005975]. A minimum number of transcripts were observed to be involved in the lipid biosynthetic process [GO:0008610]. The DE of a large number of genes linked to carbohydrate metabolism may be due to the fact that pearl millet leaves have very efficient photosynthetic and photosynthate transportation systems. It leads to the accumulation of a high content of carbohydrate (~70%–75%) in the grains. The genes linked to the lipid biosynthetic process were very low in spite of the established fact that the pearl millet grain has a very high content of TAGs (~6%–7%). This may be due to the fact that samples in the present investigation were collected during the grain-filling stage, a developmental phase in which the plant predominantly allocates resources to starch synthesis rather than lipid biosynthesis (Sreenivasulu et al., 2006). Other than the carbon assimilatory pathway, the secondary metabolic pathway, especially the phenylpropanoid pathway was observed to be most triggered in pearl millet under stress (Shivhare et al., 2020).

GO analysis of annotated transcripts based on cellular components showed a maximum of 33,173 transcripts to be associated with the integral component of the membrane [GO:0016021] followed by 19,163 transcripts with nucleus [GO:0005634] and 7,669 transcripts with cytoplasm [GO:0005737]. A minimum number of transcripts were observed to be associated with amyloplast [GO:0009501].

### Transcripts expressing unique proteins in the grains of pearl millet

The identified transcripts generated through a *de novo* transcriptomic approach were further characterized for their unique appearance in landraces (Chadhi Bajri and Damodhar Bajri), hybrid (Pusa-1201), and composite (PC-701) of pearl millet. In the case of Chadhi Bajri, we observed the appearance of sugar/inositol transporter domain-containing protein (TRINITY_DN100083_c0_g1), similar to root hairless 1 (TRINITY_DN10016_c0_g1) and caleosin-related family protein (TRINITY_DN100212_c0_g1), among others (**Supplementary Table S8**). Similarly, Damodhar Bajri showed the presence of the BTB/POZ domain-containing protein (TRINITY_DN100005_c0_g1) and E3-ubiquitin ligase (TRINITY_DN100043_c0_g1), similar to GTP-binding protein (TRINITY_DN100058_c0_g1), among others, which were uniquely expressed in Damodhar Bajri and not observed in other landraces, hybrids, and composites of pearl millet. Similarly, 3,489 unique protein-coding transcripts were reported in the Indian barnyard millet (Jayakodi et al., 2019).

Hybrids of pearl millet are quite popular and demanding among the farmers because of their desirable traits and high yield, as compared to landraces. In the case of Pusa-1201, we observed the expression of unique proteins like wall-associated kinase-like protein (TRINITY_DN100329_c0_g1), transcriptional coactivator/pterin dehydratase family protein (TRINITY_DN100329_c0_g2), and F-box associated interaction domain-containing protein (TRINITY_DN100396_c0_g1) (**Table S3**). Similarly, PC-701 showed the expression of unique proteins like conserved hypothetical protein (TRINITY_DN100004_c0_g1), zinc finger POZ domain protein (fragment) (TRINITY_DN100388_c0_g1), and galactosyl transferase family protein (TRINITY_DN100575_c1_g1). These unique proteins, as annotated through their respective transcripts, provide a specific trait to the landraces (Chadhi Bajri and Damodhar Bajri), hybrid (Pusa-1201), and composite (PC-701). This is the reason behind the unique characteristics of these genetically diverse genotypes of pearl millet.

### Identification of genes associated with rancidity/off-odor development

Rancidity/off-odor development in pearl millet flour has been considered as one of the major problems in its utilization, especially for the processing industries. Very limited work has been done in the past for the identification of genes/enzymes associated with rancidity/degradation of macromolecules in the flour.

### Identification of putative lipase transcripts

LIP is considered the first enzyme of the rancidity pathway, catalyzing the hydrolysis of triacylglycerol into FFAs and glycerol. These FFAs are subsequently acted upon by downstream enzymes, leading to the formation of volatile compounds responsible for off-odors in flour (Kumar et al., 2021). In the present study, we identified 2,038 transcripts showing homology with LIP based on conserved domains (**Supplementary Table S9**). Representative putative LIP transcripts are presented in **Table 2**, with transcript sizes ranging from 275 to 937 bp. The majority of LIP transcripts were mapped to chromosome 8, followed by chromosomes 4 and 10, consistent with the distribution of LIP gene families reported in cereals (Lee and Park, 2019). Functional annotation revealed that these transcripts belong to diverse LIP classes, including GDSL-LIP, LIP class III, monoglyceride LIP, esterase/LIP, triacylglycerol LIP, and phospholipase. We have further cloned and characterized eight LIP genes from pearl millet (Kumar et al., 2025). The identified transcripts need validation, cloning, and biochemical characterization to confirm their roles in rancidity and to explore their potential as molecular markers in future pearl millet breeding programs. Based on the accumulation of FFAs in highly rancid lines, LIP was proclaimed as the real culprit for rancidity (Aher et al., 2022).

**Table 2:** List of different variants of putative lipase (*LIP*), lipoxygenase (*LOX*), peroxidase (*POX*), and polyphenol oxidase (*PPO*) genes identified in pearl millet landraces, hybrid, and composite genotypes through *de novo* transcriptome analysis.

Trinity gene ID	Rice transcript ID	Transcript start	Transcript end	Transcript length	*E*-value	Chr. loc.	Description
Lipase genes							
TRINITY_DN100592_c0_g1	Os05t0363100-01	226	265	351	5.8	5	Similar to monoglyceride lipase
TRINITY_DN100603_c0_g1	Os04t0509100-00	430	509	799	2.11E−24	4	Similar to triacylglycerol lipase
TRINITY_DN100603_c1_g1	Os04t0509100-00	503	605	799	3.52E−37	4	Similar to triacylglycerol lipase
TRINITY_DN100894_c0_g1	Os01t0651800-01	268	402	420	1.03E−70	1	Lipase, class 3 family protein
TRINITY_DN100918_c0_g1	Os01t0315600-01	12	43	158	2.5	1	Similar to monoglyceride lipase
TRINITY_DN101215_c0_g1	Os01t0315600-01	88	113	158	0.25	1	Similar to monoglyceride lipase
TRINITY_DN101273_c0_g1	Os05t0408300-01	6	123	471	1.49E−18	5	Similar to lipase
TRINITY_DN102645_c0_g1	Os01t0521400-01	107	158	464	4.9	1	Similar to lipase family protein
TRINITY_DN103245_c0_g1	Os01t0651800-01	215	359	420	8.81E−63	1	Lipase, class 3 family protein
TRINITY_DN103245_c0_g1	Os01t0651800-01	17	359	420	1.47E−137	1	Lipase, class 3 family protein
TRINITY_DN10352_c0_g1	Os01t0521400-01	198	222	464	4.4	1	Similar to lipase family protein
TRINITY_DN104419_c0_g1	Os04t0489100-01	207	226	788	8.1	4	Lipase, class 3 family protein
TRINITY_DN104766_c0_g1	Os05t0408300-01	198	271	471	0.03	5	Similar to lipase
TRINITY_DN104958_c1_g1	Os09t0247600-01	238	275	356	1	9	Lipase, GDSL domain-containing protein
TRINITY_DN105376_c0_g1	Os12t0554500-00	291	305	610	2.9	12	Lipase, class 3 family protein
TRINITY_DN10594_c0_g1	Os04t0509100-00	63	92	799	3.7	4	Similar to triacylglycerol lipase
Lipoxygenase gene							
TRINITY_DN10043_c1_g1	Os04t0447100-02	835	908	922	4.65E−18	4	Similar to lipoxygenase
TRINITY_DN101745_c0_g1	Os11t0575600-01	87	125	868	1.1	11	Similar to lipoxygenase (fragment)
TRINITY_DN101745_c0_g1	Os11t0575600-01	87	125	868	1.2	11	Similar to lipoxygenase (fragment)
TRINITY_DN10244_c0_g1	Os08t0509100-01	805	859	941	4.3	8	Similar to lipoxygenase (EC 1.13.11.12)
TRINITY_DN105535_c0_g1	Os03t0700400-01	399	431	866	0.58	3	Lipoxygenase-3
TRINITY_DN105618_c0_g1	Os10t0361000-01	118	156	164	1.4	10	Lipoxygenase, LH2 domain protein
TRINITY_DN106988_c0_g2	Os12t0559200-02	606	679	751	1.8	12	Lipoxygenase (EC 1.13.11.12)
TRINITY_DN111005_c0_g1	Os03t0738600-01	316	389	870	3.13E−36	3	Lipoxygenase, seed germination
TRINITY_DN112458_c0_g1	Os03t0700400-01	721	739	866	3.2	3	Lipoxygenase-3
TRINITY_DN115056_c0_g1	Os08t0509100-01	574	599	941	1.5	8	Similar to lipoxygenase
TRINITY_DN117056_c0_g1	Os03t0700400-01	209	236	866	4.1	3	Lipoxygenase-3
TRINITY_DN117510_c0_g1	Os03t0700400-01	725	749	866	4.7	3	Lipoxygenase-3
TRINITY_DN117510_c0_g1	Os03t0700400-01	725	749	866	4	3	Lipoxygenase-3
TRINITY_DN118950_c0_g1	Os08t0509100-01	531	563	941	1.4	8	Similar to lipoxygenase
TRINITY_DN120868_c0_g1	Os02t0194700-01	421	447	926	1.5	2	Similar to lipoxygenase 3 (LOX2:Hv:3)
TRINITY_DN125228_c1_g2	Os05t0304600-01	769	814	847	0.6	5	Similar to lipoxygenase (fragment)
TRINITY_DN128120_c0_g1	Os03t0700400-01	45	69	866	1.10E−04	3	Lipoxygenase-3
Peroxidase genes							
TRINITY_DN10070_c0_g1	Os02t0236600-01	23	43	321	5.1	2	Peroxidase P7 (EC 1.11.1.7) (TP7)
TRINITY_DN100859_c1_g1	Os07t0638600-00	192	274	441	8.50E−25	7	Similar to class III peroxidase 44
TRINITY_DN100972_c0_g1	Os08t0549100-01	138	193	291	4.41E−19	8	Similar to ascorbate peroxidase
TRINITY_DN10118_c0_g1	Os03t0368000-00	53	83	323	4.3	3	Similar to peroxidase 1
TRINITY_DN101240_c0_g1	Os04t0689000-01	23	84	338	5.21E−21	4	Similar to peroxidase (EC 1.11.1.7)
TRINITY_DN101291_c0_g1	Os09t0323900-00	30	60	93	1.8	9	Similar to class III peroxidase 120
TRINITY_DN101628_c0_g2	Os06t0185966-00	350	406	878	3.7	6	Similar to glutathione peroxidase
TRINITY_DN102471_c0_g1	Os03t0121200-03	54	104	286	0.78	3	Similar to peroxidase 1
TRINITY_DN102593_c0_g1	Os07t0639000-01	114	168	327	4.8	7	Similar to class III peroxidase 46
TRINITY_DN1027_c0_g1	Os12t0112000-01	259	327	327	2.68E−38	12	Similar to peroxidase precursor
TRINITY_DN1027_c0_g2	Os11t0112400-01	34	315	324	4.8	11	Peroxidase (EC 1.11.1.7)
TRINITY_DN1027_c0_g2	Os12t0112000-01	37	132	327	1.38E−42	12	Similar to peroxidase precursor
TRINITY_DN1027_c0_g2	Os11t0112400-01	221	315	324	9.25E−56	11	Peroxidase (EC 1.11.1.7)
TRINITY_DN1027_c0_g2	Os11t0112400-01	34	199	324	1.39E−84	11	Peroxidase (EC 1.11.1.7)
TRINITY_DN1027_c0_g2	Os12t0112000-01	37	318	327	5.1	12	Similar to peroxidase precursor
TRINITY_DN1027_c0_g3	Os10t0107000-00	122	339	339	1.41E−96	10	Similar to class III peroxidase 124
Polyphenol oxidase genes							
TRINITY_DN112679_c0_g1	Os04t0624500-01	274	313	570	3.6	4	Similar to polyphenol oxidase
TRINITY_DN143621_c0_g1	Os04t0624500-01	331	379	570	0.16	4	Polyphenol oxidase
TRINITY_DN167165_c0_g1	Os04t0624500-01	241	310	570	4.93E−16	4	Similar to polyphenol oxidase
TRINITY_DN176233_c0_g1	Os04t0624500-01	358	398	570	2.8	4	Polyphenol oxidase
TRINITY_DN183701_c0_g1	Os04t0624500-01	227	265	570	1.06E−06	4	Similar to polyphenol oxidase
TRINITY_DN30606_c0_g1	Os04t0624500-01	338	416	570	0.005	4	Polyphenol oxidase
TRINITY_DN3256_c1_g1	Os04t0624500-01	233	260	570	7.8	4	Polyphenol oxidase
TRINITY_DN55450_c0_g2	Os04t0624500-01	61	95	570	3.2	4	Polyphenol oxidase
TRINITY_DN57292_c0_g1	Os04t0624500-01	302	333	570	1.8	4	Polyphenol oxidase
TRINITY_DN59833_c0_g1	Os04t0624500-01	168	194	570	3.8	4	Similar to polyphenol oxidase
TRINITY_DN64284_c0_g1	Os04t0624500-01	118	570	570	3.5	4	Polyphenol oxidase
TRINITY_DN6503_c0_g1	Os04t0624500-01	373	428	570	1.89E−17	4	Polyphenol oxidase
TRINITY_DN6503_c0_g2	Os04t0624500-01	80	570	570	0	4	Polyphenol oxidase

### Identification of putative lipoxygenase transcripts

Lipoxygenase uses FFAs as a substrate and form hydroperoxide and many other carbonyl compounds like aldehydes, alcohols, and ketonyl compounds. These products basically act as a substrate for the off-odor development in the flour (Sinha et al., 2020). LOX has a small N-terminal PLAT domain and a major C-terminal catalytic domain that were used in the present investigation to find out the transcripts showing homology with LOX. We have identified 209 putative transcripts showing homology with LOX reported from pearl millet (**Supplementary Table S10**). The size of the identified LOX transcripts was observed in the range of 125 to 941 bp. Most of the identified putative LOX transcripts were observed lying on Chr 3 followed by Chr 2 and Chr 4. The identified LOX was observed to be associated with different families like LOX2.3, LOX-3, and LOX LH2 domain-containing protein. Furthermore, we have cloned two LOX genes from pearl millet (*LOX*—accession no. OQ184873, *LOX-6*—accession no. OQ184874). In-depth kinetics study of these enzymes will help in regulating their activity and developing technology for enhancing the shelf life of pearl millet flour. Twelve LOX genes were identified from foxtail millet showing differential expression in response to different biological processes (Zhang et al., 2021).

### Identification of putative hydroperoxide lyase transcripts

The hydroperoxide formed due to the activity of LOX enzyme on FFAs is used as a potential source for the production of carbonyl compounds like aldehyde, alcohol, and ketonyl by the HPL enzyme. Genes/proteins of this enzyme have not been characterized much in pearl millet. We have identified 26 transcripts showing homology with HPL (**Supplementary Table S11**). The size of the identified HPL genes was observed in the range of 480 to 510 bp. All the identified *HPL* was observed localized on Chr 2. Different variants of *HAL* have been identified and reported from different crops like soybean and rye. Many HPL genes have been identified and cloned from grape berries and characterized to play an important role in sugar metabolism and aroma volatiles (Šikuten et al., 2025).

### Identification of putative peroxidase transcripts

POX is considered as a very versatile monomeric glycoprotein having two prominent domains—the heme group and the ferriprotoporphyrin prosthetic group. This enzyme is involved in different processes like detoxifying, neutralizing ROS, and producing various ketonyl compounds. Here, we identified 1,023 transcripts showing homology with POX from pearl millet (**Supplementary Table S12**). The size of the identified transcripts was in the range of 93–878 bp. The identified POX transcripts were observed localized on almost all the chromosomes with a maximum on Chr 7 followed by Chr 3 and Chr 4. Ma et al. (2022) identified 132 genes of class III POX in the whole genome of foxtail millet.

### Identification of polyphenol oxidase transcripts

PPO catalyzes the oxidation of phenols to quinone, which in turn imparts a brown/dark color to the product. Here, we have identified 17 transcripts showing homology with the PPO gene based on domain search analysis and were observed to be lying on Chr 4 (**Supplementary Table S13**). Niranjan-Raj et al. (2019) identified and cloned a putative PPO gene of 395 bp from pearl millet. Four PPO genes were reported from virgin olive oil and one gene was characterized to play a role in catalyzing the hydroxylation of tyrosol to form hydroxytyrosol (Sánchez et al., 2023).

### Identification of genes associated with starch degradation

Starches constitute a major share of nutrient composition of pearl millet grain and are stored in complex form inside the endospermic tissue. Various isoforms of amylases are involved in the degradation of starch forming maltose and simple sugars. It also invites various other pathogenic microorganisms compromising the quality of the flour. Here, we have identified 401 transcripts showing homology with different variants of amylases (**Supplementary Table S14**). The sizes of the transcripts were in the range of 244–1,121. Most of the genes were observed localized on Chr 8 followed by Chr 9 and Chr 7. Some of the variants of the amylases identified are glucoamylase, starch debranching enzyme, and α/β-amylases. Yadav et al. (2022) identified a single α/β-amylase gene from pearl millet through a genome-wide association study (GWAS).

### Validation of RNA-seq data using qRT-PCR

**Figure 3:**
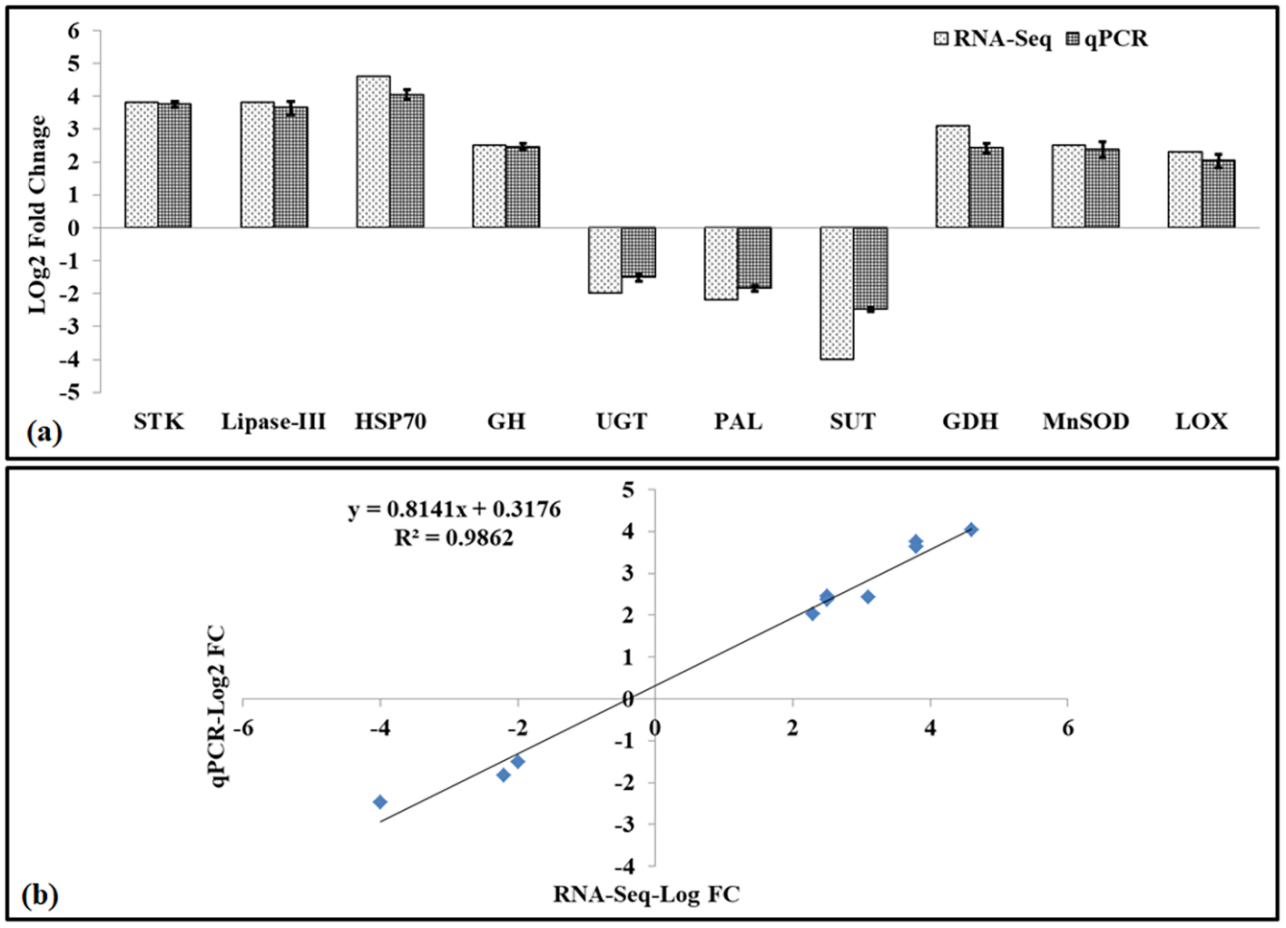
Validation of RNA-seq data using quantitative real-time PCR (qRT-PCR). **(a)** Log_2_ fold change comparison of RNA-seq (dotted bars) and quantitative real-time PCR (qRT-PCR, netted bars) of 10 differentially expressed genes (DEGs). **(b)** Correlation between RNA-seq and RT-PCR gene expression (*R*^2^ = 0.9862, *p*<0.05); lines above the qRT-PCR bar indicate a significant difference between treatments (*p* ≤ 0.05, one-way ANOVA).

To validate RNA-seq data, we have randomly selected 10 DETs and used their digital fold expression (log_2_ FC) from the RNA-seq dataset for comparative analysis with relative fold change (converted to log_2_ FC), as achieved through qPCR (**Figure 3**).

The DETs selected for the comparative analysis were Serine/threonine kinase (*STK*, DN2506), Lipase class-III (*Lip-III*, DN63949), Heat shock protein 70 (*HSP70*, DN1582), Glucoside hydrolase (*GH*, DN15277), UDP glucosyltransferase (*UGT*, DN51146), Phenylalanine lyase (*PAL*, DN8260), Sucrose transporter (*SUT*, DN48102), Glutamate dehydrogenase (*GDH*, DN254), Mn-superoxide dismutase (*MnSOD*, DN131007), and Lipoxygenase (*LOX*, DN7868). The fold expression (2^−ddCt^ value) was converted to log_2_ fold change for comparative analysis with RNA-seq dataset and for establishing the correlation between the RNA-seq and qPCR datasets. We observed significant variations (*p* ≤ 0.05) in the expression (upregulation and downregulation) of respective DEGs, as observed through RNA-seq data and qPCR data (**Figure 3**). A strong correlation (*R*^2^=0.98) was established between the RNA-seq dataset and qPCR dataset, which validates the quality of the data generated using the RNA-seq.

### Expression analysis of genes linked to flour rancidity

Different variants of genes, as predicted through a domain-based homology search from *de novo* transcriptome sequencing data and few cloned in our lab, were used for the expression analysis: Lipase gene [*Lip-1* (accession no. OQ184871), *Lip-2* (accession no. MZ590565), *Lip-3* (accession no. MZ590564), and *Lip-4* (accession no. OQ304605)], Lipoxygenase [*LOX-1* (accession no. OQ184873), *LOX-2* (accession no. OQ184874), *LOX-3* (TRINITY_DN10043_c0_g1_i1), and *LOX-4* (TRINITY_DN118950_c0_g1)], Peroxidase [*POX-1* (TRINITY_DN109983), *POX-2* (TRINITY_DN177283), *POX-3* (accession no. PP171489), and *POX-4* (TRINITY_DN115862)], and Polyphenol oxidase [*PPO-1* (accession no. PP765147), *PPO-2* (TRINITY_DN183701_c0_g1), *PPO-3* (TRINITY_DN167165_c0_g1), and *PPO-4* (TRINITY_DN112679_c0_g1)].

**Figure 4:**
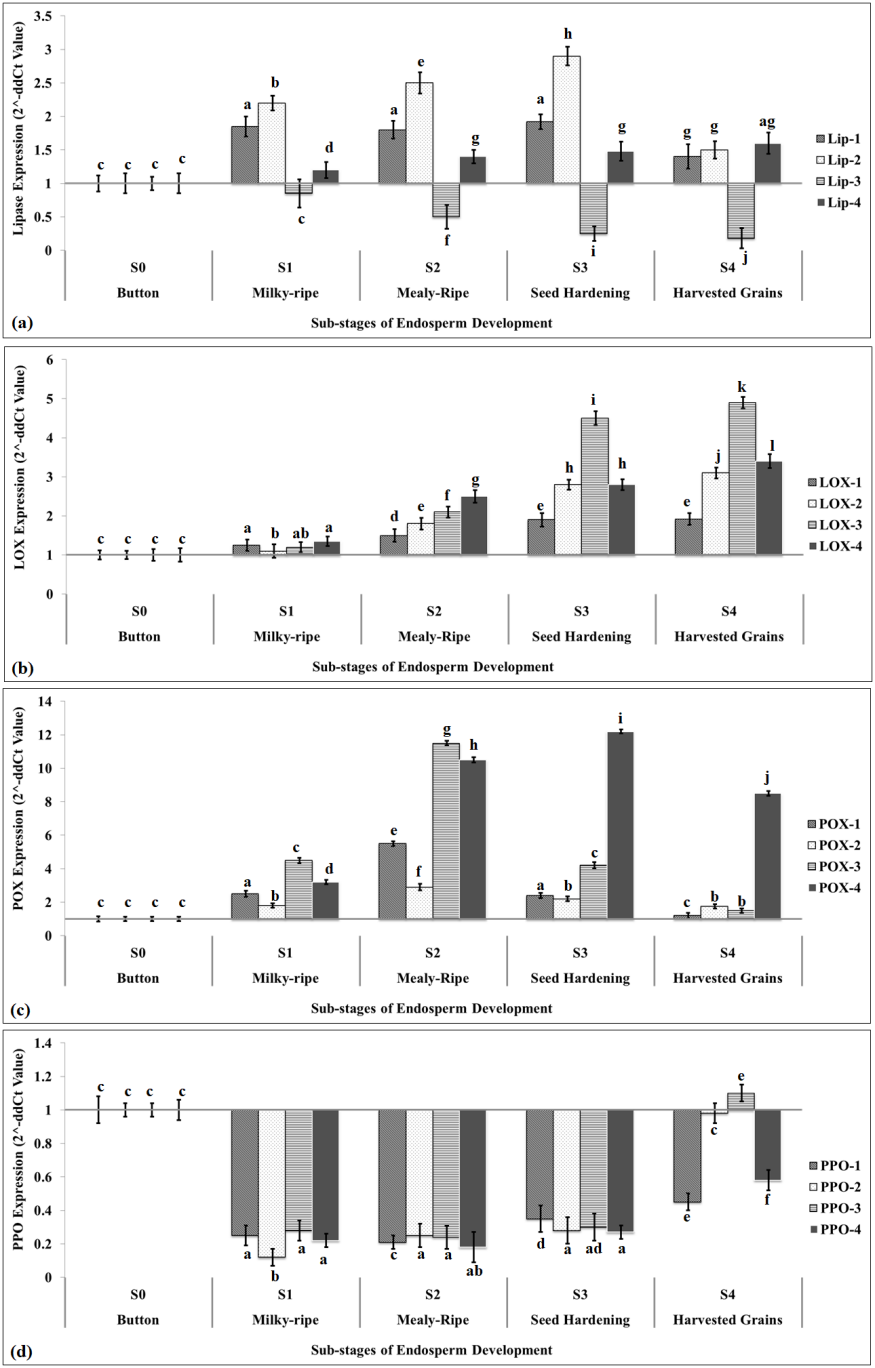
Tissue-specific expression analysis of identified rancidity-causing putative genes in endosperm of pearl millet using quantitative real-time PCR. **(a)** Expression of lipase gene, **(b)** expression of LOX gene, **(c)** expression of POX gene, and **(d)** expression of PPO gene. Sub-stages of endosperm used for the expression analysis: S_0_, button stage; S_1_, milky-ripe; S_2_, mealy-ripe; S_3_, seed hardening; S_4_, harvested grains; four different variants of *LIP*, *LOX*, *POX*, and *PPO* were used for the expression analysis; Actin-7 gene (acc. no. KM105957.1) was used as endogenous control; all data are presented as mean ± SE of three replicates; lines above each bar indicate a significant difference between treatments (*p* ≤ 0.05, one-way ANOVA).

We have identified four LIP (*Lip-1*, *Lip-2*, *Lip-3*, and *Lip-4*) genes to be used for the tissue-specific expression analysis. *Lip-2* showed maximum relative fold expression (2.95-fold) during the S_3_ stage (seed hardening), as compared to the S_1_ stage (button stage) (**Figure 4a**). *Lip-3* showed downregulation throughout the different sub-stages of endospermic tissue growth with minimum expression in harvested grains. All the *Lip* genes identified showed a significant (*p* ≤ 0.05) increase in the relative fold expression at different sub-stages of endospermic tissue growth with maximum expression during the seed hardening stage. The expression of *Lip* variants was observed to be very low in harvested grains, as compared to the button stage.

We also identified four different variants of LOX genes (*LOX-1*, *LOX-2*, *LOX-3*, and *LOX-4*) and evaluated their tissue-specific expression pattern. *LOX-3* showed maximum relative fold expression (5.1-fold) during the seed harvesting stage, as compared to the button stage (**Figure 4b**). We observed a significant (*p* ≤ 0.05) increase in the expression of all the four *LOX* variants at different stages of endospermic tissue growth and development. The abundance of transcript was observed minimum in the case of *LOX-1* at different sub-stages of endospermic tissue growth compared with other variants of *LOX*.

We identified four different variants of POX (*POX-1*, *POX-2*, *POX-3*, and *POX-4*) genes and captured their expression pattern during the growth and development of endospermic tissue. Expression analysis showed maximum relative fold expression of *POX-4* (12.1-fold) in harvested grains, as compared to the button stage (**Figure 4c**). We observed an abundance of transcripts of all the four variants of *POX* during different sub-stages of endosperm growth and development, except harvested grains, where the *POX-4* transcript was observed at a maximum.

We selected four variants of PPO (*PPO-1*, *PPO-2*, *PPO-3*, and *PPO-4*) genes and captured their expression pattern in developing endosperm tissue of pearl millet. All the variants of *PPO* showed significant downregulation at different sub-stages of developing endosperm, as compared to the button stage (**Figure 4d**). *PPO-3* showed upregulation (1.15-fold) in the harvested grain, as compared to the button stage. *PPO-2* was observed to be highly downregulated during the milky-ripe stage (−0.1-fold), as compared to other variants of *PPO*. Although much information is not available on the expression pattern of these rancidity-causing genes in millets, some literature reported the DE of these genes in different crop species under other environmental factors. The tissue-specific expression pattern of 21 LOX genes was reported in *Cannabis sativa* (Fayaz et al., 2023).

### Activity assay of rancidity-causing enzymes

We have observed four prominent enzymes (LIP, LOX, POX, and PPO) to be involved in off-odor development and lipid hydrolysis in pearl millet flour. The activities of all the four enzymes were analyzed in samples stored at different durations (0, 10, 30, 60, and 90 DAM).

**Figure 5:**
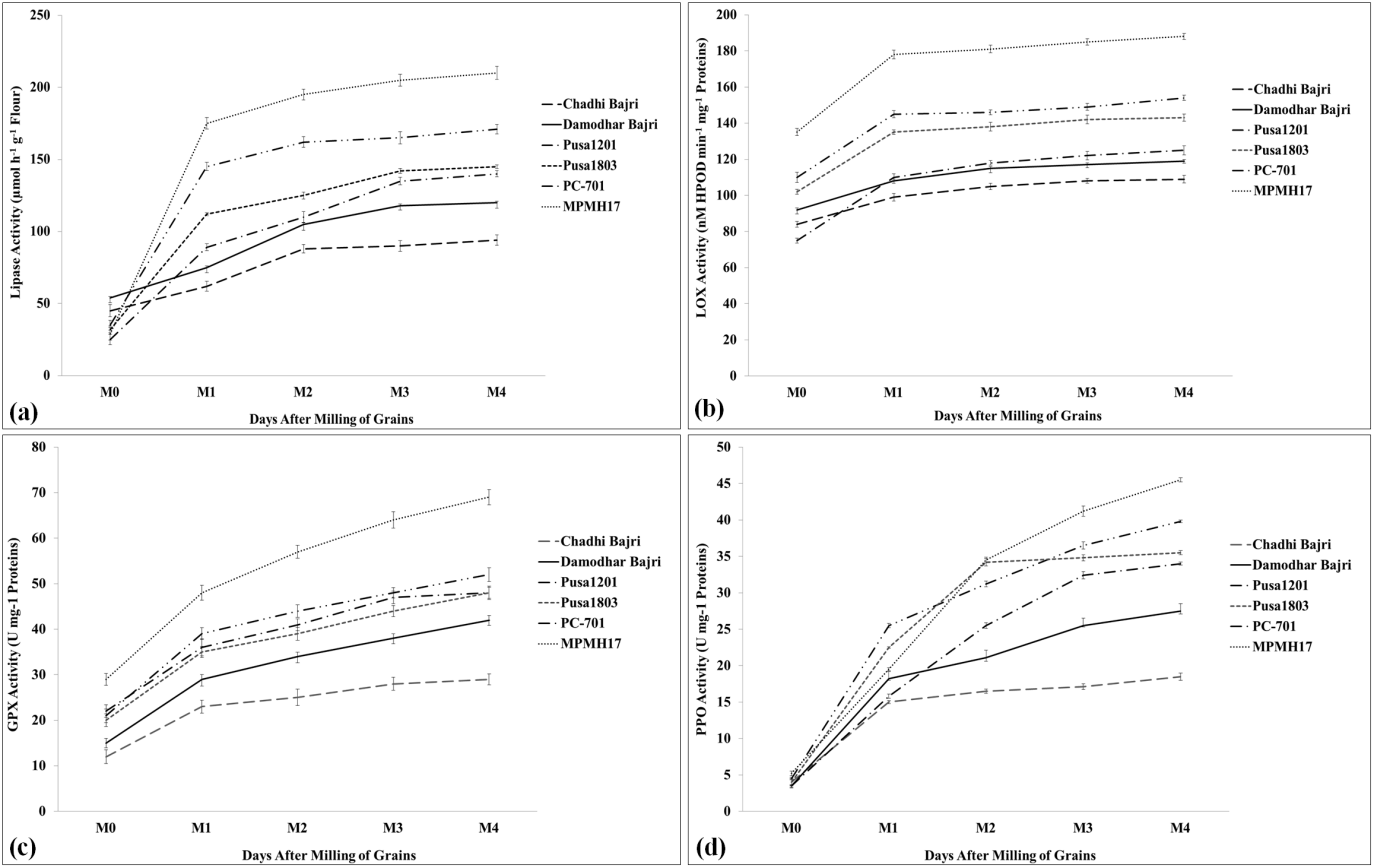
Activity assay of rancidity-causing enzymes in diverse genotypes of pearl millet. **(a)** Activity assay of lipase, **(b)** activity assay of lipoxygenase, **(c)** activity assay of guaiacol peroxidase, and **(d)** activity assay of polyphenol oxidase; six diverse genotypes were selected for the activity assay. Days after milling (DAM) of grains: M_0_, 0 DAM; M_1_, 10 DAM; M_2_, 30 DAM; M_3_, 60 DAM; M_4_, 90 DAM; all data are presented as mean ± SE of three replicates; lines above each bar indicate a significant difference between treatments (*p* ≤ 0.05, one-way ANOVA).

LIP activity was observed to be at a minimum (88.5 µmol h^−1^ g^−1^ flour) in the flour of pearl millet cv. Chadhi Bajri stored for 90 DAM; a non-significant increase in the LIP activity was observed from 10 DAM onward (**Figure 5a**). The LIP activity was observed to be at a maximum (200.5 µmol h^−1^ g^−1^ flour) in cv. MPMH17 stored for 90 DAM. We observed a significant (*p* ≤ 0.05) increase in the activity of LIP enzyme, until M_1_ (10 DAM) in all the selected contrasting genotypes, and further non-significant (*p* ≤ 0.05) variations were observed with an increase in the number of DAM except cv. MPMH17. A similar pattern of LIP activity was observed in the flour of diverse germplasm of pearl millet stored at different durations (Goswami et al., 2020).

LOX assay showed minimum activity in the flour of pearl millet cv. Damodhar Bajri stored at different durations. The activity was observed to be at a minimum (103 nM HPOD min^−1^ mg^−1^ protein) during the M_4_ stage (90 DAM). The LOX activity was observed to be at a maximum (184 nM HPOD min^−1^ mg^−1^ protein) in pearl millet cv. Pusa-1803 during the M_4_ stage (90 DAM) (**Figure 5b**). Overall, the LOX activity was significantly (*p* ≤ 0.05) maximum in cv. Pusa-1803 analyzed in a sample stored at different durations, as compared to other contrasting *cvs*. We observed significant (*p* ≤ 0.05) variations in the LOX activity in samples stored up to 10 DAM, and further non-significant changes were observed in the stored flour up to 90 DAM. The findings are in conformity with the observation made by Goswami et al. (2020) and Kumar et al. (2025) in pearl millet.

A similar pattern of GPX activity was observed in the samples stored at different durations with maximum activity in the flour of cv. Pusa 1803 and minimum activity in cv. Damodhar Bajri. Pusa-1803 showed a gradual increase in the GPX activity until 90 DAM (**Figure 5c**). A very high activity of POX was observed in stored flour of pearl millet, and it was reported to be used as one of the biochemical markers for analyzing the shelf life of flour (Goyal et al., 2017).

PPO assay showed a very significant (*p* ≤ 0.05) increase in the activity during the first 10 DAM in all the selected *cvs*. The PPO activity was observed to be at a minimum in cv. Damodhar Bajri estimated at different time points (DAM), as compared to other *cvs*. The activity was observed to be at a maximum in cv. Pusa-1803 during M_4_ (90 DAM) and we observed an increased trend of PPO activity throughout the storage period (**Figure 5d**). A similar pattern of LIP, LOX, POX, and PPO activities was reported in the flour of contrasting pearl millet *cvs* (Kumar et al., 2024).

### Remodeling the rancid pathway

**Figure 6:**
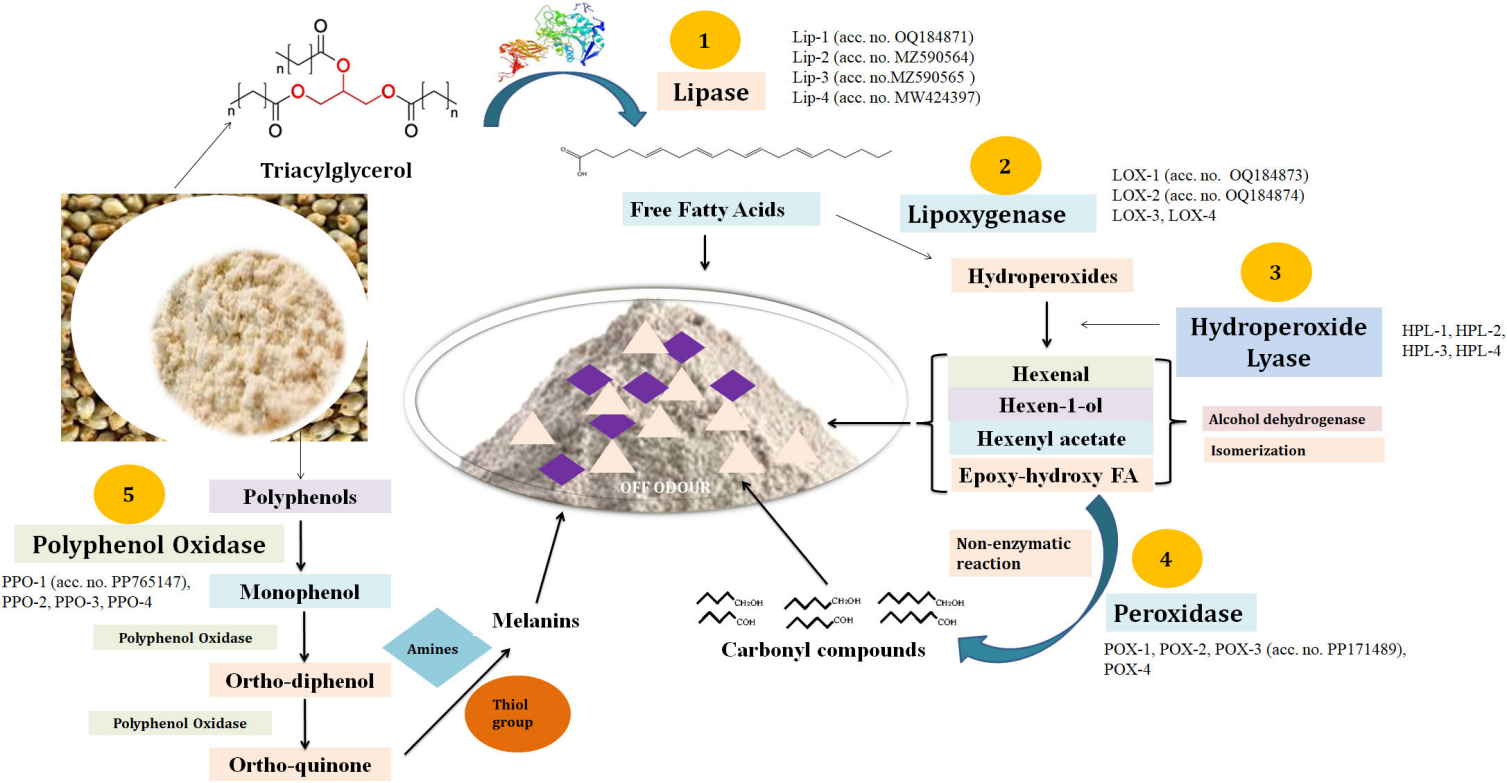
Molecular mechanism of rancidity development in pearl millet flour. The schematic integrates key genes, enzymes, and metabolites identified through transcriptomic and biochemical analyses: (1) Lipases (Lip 1–4) hydrolyze triacylglycerols (TAGs) into free fatty acids (FFAs); (2) lipoxygenases (LOX 1–4) oxidize polyunsaturated FFAs to hydroperoxides; (3) hydroperoxide lyases (HPLs) and peroxidases (POX 1–4) convert hydroperoxides into volatile aldehydes/ketones (hexanal, nonanal); and (4) polyphenol oxidases (PPO 1–4) catalyze phenolic oxidation, contributing to discoloration. Gray arrows indicate enzymatic reactions; colored boxes highlight differentially expressed genes (red: upregulated in high-rancid genotypes; blue: downregulated).

Pearl millet has a very high content of lipid (~6%–7%) along with other beneficial nutrients in the grains. The pearl millet grains have numerous compartments packed with different macro- and micronutrients and catabolic enzymes responsible for rancidity (Aher et al., 2022; Kumar et al., 2025). The increase in the temperature upon milling triggered the activities of catabolic enzymes like α/β-amylases, proteases, and LIPs. This leads to the degradation of key nutrients accumulated inside the grains through oxidative/hydrolytic reactions (Ali et al., 2023a). We have identified almost 2,038 transcripts showing homology with LIP (**Figure 6**). Furthermore, we cloned eight LIP genes (accession nos. OQ184871, MZ590565, MZ590564, OQ304605, OQ304606, MW424397, PP107929, and PP114100) from contrasting pearl millet genotypes, and four were reported to be localized in the grains.

The product of triacylglycerol (TAG) degradation is FFAs and glycerol. The FFAs formed are further acted upon by LOX, which is a very stable, dynamic, and versatile enzyme (**Figure 6**). It acts on FFAs and forms hydroperoxides. We have identified 209 transcripts showing homology with the LOX gene and cloned two LOX genes (accession nos. OQ184873 and OQ184874) observed to be localized in the seeds. The hydroperoxides formed are further acted upon by the fatty acid HPL and forms short-chain aldehydes and oxo-acids like 3-hexenal, 11-formyl-9-undecenoic acid, and octanoic acid. These aldehydes, oxyacids, and ketonyl compounds are basically responsible for the off-odor development in pearl millet flour. We have identified 26 HPL genes in the present investigation and observed four to be localized in the endospermic tissue. We have not cloned any HPL gene from pearl millet. The POX enzyme acts on hydroperoxides and forms various carbonyl compounds mainly responsible for the off-odor and discoloration of the flour (**Figure 6**). We have identified 1,023 transcripts showing homology with the POX gene and cloned the putative POX-III gene (accession no. PP171489) from pearl millet cv. Pusa-1201 and was observed to be localized in the developing endosperm.

The pearl millet grain has an abundance of unique polyphenols that have numerous health benefits. Upon milling, these polyphenols are oxidized by the PPO enzyme and form a quinone compound that gives a brown/dark color to the flour upon storage. Here, we identified 17 putative transcripts showing homology with *PPO* and cloned a putative PPO gene (accession no. PP765147) from pearl millet cv. Pusa-1201. *In silico* characterization showed the respective protein to be localized in the grains. Combined metabolomics and transcriptomics showed the over-expression of SDR, FATA, FATB, and MFP responsible for the rancidity of FFAs in oil palm fruit (Zhang et al., 2023).

Overall, we predicted the genes responsible for the synthesis of rancidity-causing enzymes, their reactions, and metabolites, leading to off-odor development in flour. The findings are in conformity with the observation of Bhunia et al. (2023) in rice bran. They predicted enzymes and pathways responsible for the hydrolytic and oxidative rancidity of rice bran lipids. LIP initiates the TAG hydrolysis, and the product of the reaction is utilized by the LOX for oxidation and production of hydroperoxides—considered as the first definitive step in off-odor development. These hydroperoxides are further metabolized by HPL, POX, and alcohol dehydrogenases into aldehydes, acids, and peroxides, which are mainly responsible for the rancidity and bitterness of flour.

This degradation cascade occurs most rapidly within the first 10 DAM, after which it gradually slows down with storage. The identification and characterization of these key genes and enzymes laid the foundation for developing targeted strategies/technologies to regulate the activities of these enzymes in order to enhance the shelf life of pearl millet flour.

## Conclusions

In conclusion, this study provides the first comprehensive *de novo* transcriptomic resource elucidating the molecular basis of rancidity in pearl millet flour. High-quality sequencing of diverse genotypes revealed over 219,000 genes and 386,000 transcripts, with differential expression analyses identifying key lipid- and starch-degrading enzymes—particularly LIPs, LOXs, POXs, and PPOs—as central drivers of post-milling deterioration. Landraces consistently exhibited lower expression of rancidity-associated genes than hybrids and composites, underscoring the potential of traditional germplasm for breeding shelf-stable varieties. Functional annotation and GO analysis linked the most enriched pathways to lipid catabolism, fatty acid oxidation, and oxidative browning, while tissue-specific expression profiling highlighted Lip-2, LOX-3, and POX-4 as major contributors during seed hardening and storage. Enzyme activity assays corroborated transcriptomic data, demonstrating early, sharp increases in hydrolytic and oxidative activities within 10 DAM, followed by sustained activity during storage. This integrative approach not only mapped the rancidity pathway—from triacylglycerol hydrolysis to aldehyde and ketone formation—but also pinpointed candidate genes and biochemical markers for targeted intervention. The results offer a genomic and biochemical foundation for pathway remodeling strategies, including breeding, genome editing, and post-harvest processing innovations, to mitigate rancidity without compromising nutritional quality. Ultimately, the findings pave the way for developing pearl millet cultivars and processing technologies with improved flour shelf life, thereby enhancing the grain’s marketability, nutritional impact, and role in food security.

## Data Availability

The original contributions presented in the study are publicly available. This data can be found here: https://www.ncbi.nlm.nih.gov/bioproject/625418/, accession No. PRJNA625418.

## Supplementary material

Supplementary Figure 1 Histogram showing the distribution of assembled transcripts length generated using *de novo* transcriptome sequencing of landraces, hybrid and composite of pearl millet.

Supplementary Figure 2 Volcano plot analysis of DEGs identified in landraces, hybrid and composite of pearl millet using *de novo* transcriptome sequencing, a) Volcano plot analysis of DEGs in Damodhar Bajri vs Chadhi Bajri, b) Volcano plot analysis of DEGs in Pusa 1201 vs Chadhi Bajri, c) Volcano plot analysis of DEGs in PC701 vs Damodhar Bajri, and d) Volcano plot analysis of DEGs in Pusa 1201 vs PC701; Red is used to represent genes which were up-regulated, and blue represent genes which were down-regulated.
