# Supplementary figures and images for: When genes turn traitor: de novo transcriptomics uncovers pearl millet’s rancidity machinery
Source: Front Plant Sci. 2025 Nov 17;16:1677082. doi: 10.3389/fpls.2025.1677082 (PMC12666563; doi:10.3389/fpls.2025.1677082)

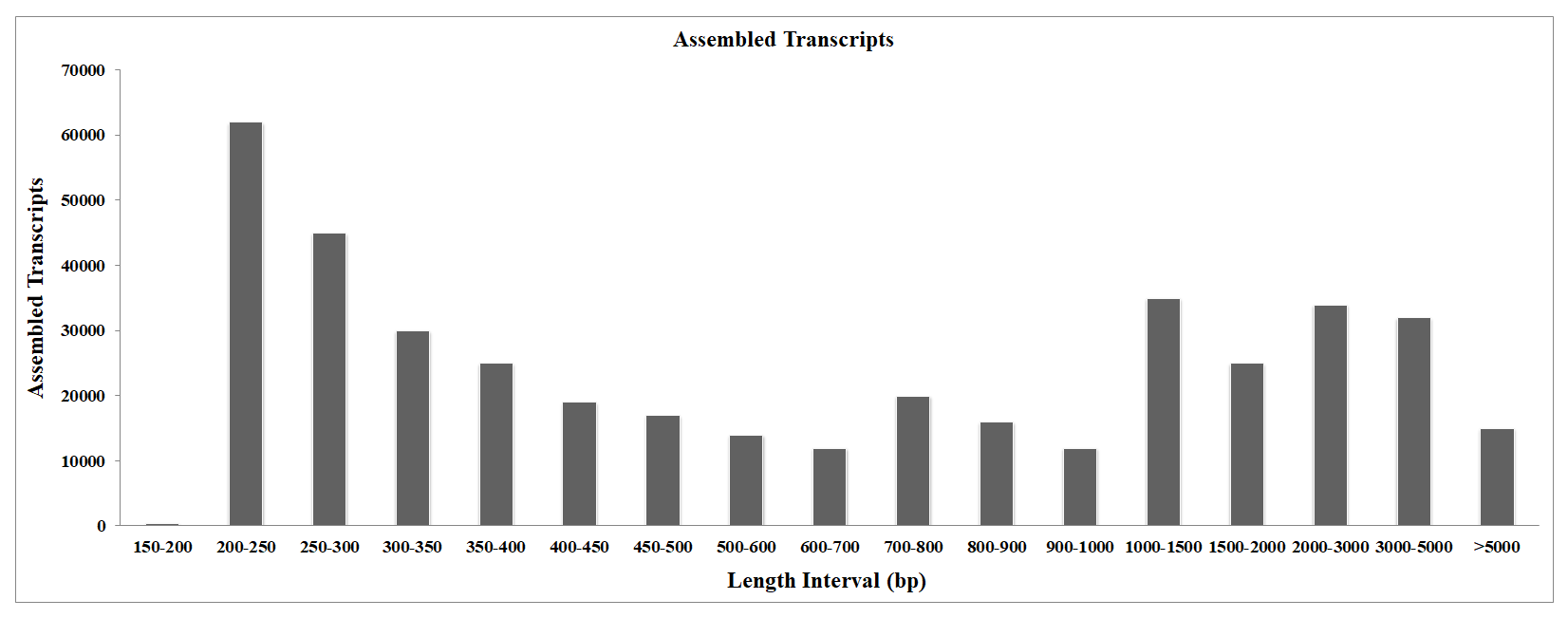

Supplement: Supplementary Figure 1 — Histogram showing the distribution of assembled transcripts length generated using de novo transcriptome sequencing of landraces, hybrid and composite of pearl millet. [file Image1.tif]

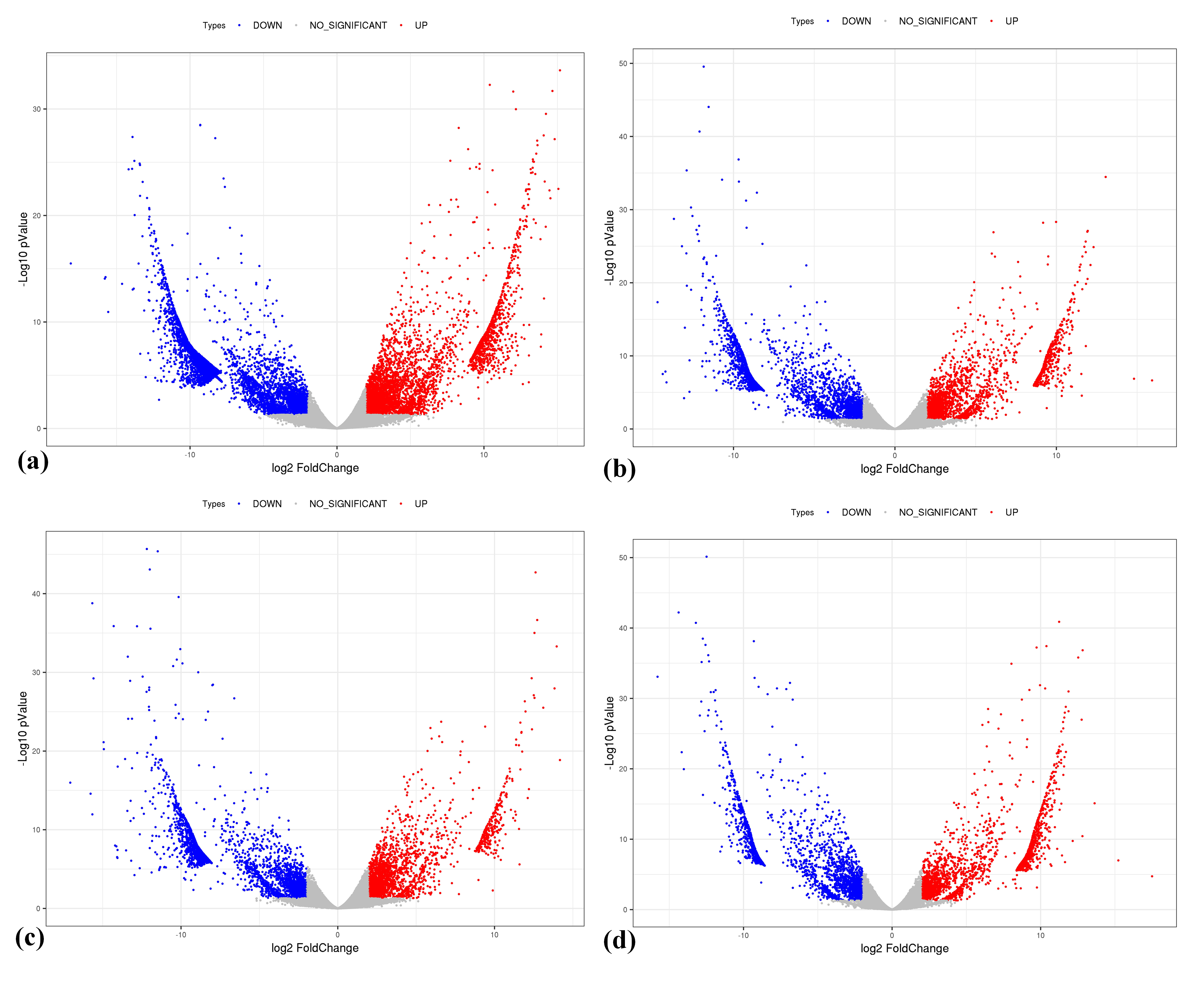

Supplement: Supplementary Figure 2 — Volcano plot analysis of DEGs identified in landraces, hybrid and composite of pearl millet using de novo transcriptome sequencing, a) Volcano plot analysis of DEGs in Damodhar Bajri vs Chadhi Bajri, b) Volcano plot analysis of DEGs in Pusa 1201 vs Chadhi Bajri, c) Volcano plot analysis of DEGs in PC701 vs Damodhar Bajri, and d) Volcano plot analysis of DEGs in Pusa 1201 vs PC701; Red is used to represent genes which were up-regulated, and blue represent genes which were down-regulated. [file Image2.tif]
