# Supplementary material for: When genes turn traitor: de novo transcriptomics uncovers pearl millet’s rancidity machinery
Source: Front Plant Sci. 2025 Nov 17;16:1677082. doi: 10.3389/fpls.2025.1677082 (PMC12666563; doi:10.3389/fpls.2025.1677082)
Supplement: Supplementary file 5 [file DataSheet3.docx]

**Table S6.** Comparative differential gene expression (DGE) analysis of annotated transcripts identified from landraces, hybrid and composite of pearl millet using de novo transcriptome sequencing.

|  | **Genes** | | **Isoforms** | |
| --- | --- | --- | --- | --- |
| **Treated vs Control** | **Downregulated** | **Upregulated** | **Downregulated** | **Upregulated** |
| Damodhar Bajri vs Chadi Bajri | 2,834 | 2,158 | 5,690 | 4,300 |
| PC701 vs Chadi Bajri | 2,811 | 2,227 | 6,226 | 4,921 |
| PC701 vs Damodhar Bajri | 1,491 | 1,895 | 3,994 | 4,754 |
| Pusa-1201 vs Chadi Bajri | 2,623 | 2,078 | 5,789 | 4,095 |
| Pusa-1201 vs Damodhar Bajri | 2,289 | 2,464 | 5,146 | 5,295 |
| Pusa-1201 vs PC701 | 2,077 | 2,186 | 5,436 | 4,941 |
