# Supplementary material for: When genes turn traitor: de novo transcriptomics uncovers pearl millet’s rancidity machinery
Source: Front Plant Sci. 2025 Nov 17;16:1677082. doi: 10.3389/fpls.2025.1677082 (PMC12666563; doi:10.3389/fpls.2025.1677082)
Supplement: Supplementary file 7 [file DataSheet5.docx]

**Table S8.** List of identified transcripts expressing unique proteins in landraces, hybrid and composite of pearl millet.

| Chadi-bajri |  |
| --- | --- |
| TRINITY_DN100083_c0_g1 | Sugar/inositol transporter domain containing protein |
| TRINITY_DN10016_c0_g1 | Similar to root hairless1 |
| TRINITY_DN100212_c0_g1 | Caleosin related family protein |
| TRINITY_DN100266_c0_g1 | Similar to NB-ARC domain containing protein |
| TRINITY_DN10054_c0_g1 | Zinc finger, BED-type predicted domain containing protein |
| DamodharBajri |  |
| TRINITY_DN100005_c0_g1 | Similar to BTB/POZ domain containing protein |
| TRINITY_DN100043_c0_g1 | E3-ubiquitin ligase, Modulation of the cold stress respons |
| TRINITY_DN100058_c0_g1 | Similar to GTP-binding protein |
| TRINITY_DN100144_c0_g1 | Similar to DNA-directed RNA polymerase I subunit 12 (EC 2.7.7.6) (Nuclear RNA polymerase I small specific subunit Rpa12) (Zinc ribbon domain containing protein 1) |
| TRINITY_DN100480_c1_g1 | Similar to Chalcone synthase C2 (EC 2.3.1.74) (Naringenin-chalcone synthase C2) |
| PC701 |  |
| TRINITY_DN100004_c0_g1 | Conserved hypothetical protein |
| TRINITY_DN100388_c0_g1 | Similar to Zinc finger POZ domain protein (Fragment) |
| TRINITY_DN100575_c1_g1 | Galactosyl transferase family protein |
| TRINITY_DN101079_c1_g1 | Succinate dehydrogenase iron-protein subunit (SDHB) |
| TRINITY_DN10109_c0_g3 | Similar to Tubulin folding cofactor B |
| Pusa-1201 |  |
| TRINITY_DN100329_c0_g1 | Similar to Wall-associated kinase-like protein |
| TRINITY_DN100329_c0_g2 | Transcriptional coactivator/pterin dehydratase family protein |
| TRINITY_DN100396_c0_g1 | F-box associated interaction domain domain containing protein |
| TRINITY_DN100483_c0_g1 | Acetolactate synthase (EC4.6.3.8), Acetohydroxy acid synthase, Herbicide resistance (mutated form of rice ALS, OsmALS (W548L/S627I) |
| TRINITY_DN100515_c0_g1 | Similar to Saccharopine dehydrogenase family protein, expressed |
