# Supplementary material for: When genes turn traitor: de novo transcriptomics uncovers pearl millet’s rancidity machinery
Source: Front Plant Sci. 2025 Nov 17;16:1677082. doi: 10.3389/fpls.2025.1677082 (PMC12666563; doi:10.3389/fpls.2025.1677082)
Supplement: Supplementary file 14 [file Table1.docx]

**Table S1.** List of primers used for the expression analysis of differentially expressed transcripts (DETs) using quantitative Real-Time PCR.

| ***Gene Name** | **Oligo ID** | **Sequence (5'-3')** | **Tm (℃)** |
| --- | --- | --- | --- |
| Serine Threonine Kinase | STK-F | GCATGTACAGCCCTATAGACAG | 58.1 |
|  | STK-R | GCCACACAAGGGTATACACA | 57.5 |
| Lipase-III | LIP-III-F | ACAACACACGAACACAATGG | 57.1 |
|  | LIP-III-R | CTTGTCTTTCAGGGCATCGA | 57.9 |
| Heat Shock Protein 70 | HSP70-F | TACTCCAAGCGAATGAACCG | 58.2 |
|  | HSP70-R | ACCCACCAACTATGATCCGA | 58.1 |
| Glucoside hydrolase | GH-F | GAAAAGCCAAGCAAGACGAC | 57.7 |
|  | GH-R | GCTTTGTACGTGCATTCCTG | 57.9 |
| UDP Glucosyltransferase | UGT-F | CAGCAACCAAGACGATCTCA | 57.8 |
|  | UGT-R | GCTCAACATTCAGCCATTCAG | 57.9 |
| Phenylalanine Lyase | PAL-F | AAGTAGACAGCAGCAACACC | 58.1 |
|  | PAL-R | TGGCACCCAGTTATTTCCTG | 57.7 |
| Sucrose Transporter | SuT-F | TTTCACGGCTAGCTCACTTC | 57.9 |
|  | SuT-R | CGCCATCCAAGAACAGAAGA | 57.9 |
| Glutamate Dehydrogenase | GDH-F | ACTCTGCCCGAAAATACGTC | 57.9 |
|  | GDH-R | GATTATCCTCGTCGATGGCC | 58.1 |
| Mn-Superoxide dismutase | MnSOD-F | CTCCAGCGTACTTCCAGTTC | 57.8 |
|  | MnSOD-R | GGAAAACCATGGCTCCTCAT | 58.1 |
| Lipoxygenase | LOX-F | CCTCGGGTGATAGGGTGTG | 59.1 |
|  | LOX-R | AACCCCAAATCCTCCGATCA | 58.7 |
| Lipase-I | LIP-1-F | TCATAGTCACAGGGCACTCA | 58.3 |
|  | LIP-1-R | CCGTGTACCAGTCGAATTGT | 57.9 |
|  | LIP-2-F | CACGCAGATCATCCAGAAGG | 58.4 |
|  | LIP-2-R | GATCATCGACCGGAAGTTGG | 58.4 |
|  | LIP-3-F | GAGAAGCTGATCATCCGGTC | 57.8 |
|  | LIP-3-R | CGCGATGAAGTTGAGGATGA | 58.1 |
|  | LIP-4-F | CAGGTTCGCTCAAATCCGTA | 58.1 |
|  | LIP-4-R | AGTTTTGTCCCTGCCTGATG | 58.3 |
| Lipoxygenase | LOX-1-F | GGACATCTCAACGGACTCAC | 58.2 |
|  | LOX-1-R | TAGCGATCGGTTTCAGTGTG | 58.1 |
|  | LOX-2-F | CATGATGCCTACCTGCCATT | 58.3 |
|  | LOX-2-R | GTTGGTGGTAGCATCAGAGG | 57.9 |
|  | LOX-3-F | GAGAACAACAACGCCGAAAC | 58.2 |
|  | LOX-3-R | CGTATTGGCCAGGTTTCGTA | 57.9 |
|  | LOX-4-F | GACATGCTTCACTTCCACCT | 57.8 |
|  | LOX-4-R | CCTTGACCAGCCTGAAAGTT | 58.2 |
| Peroxidase | POX-1-F | ACAGGCCAAGTACAAGGTTG | 58.3 |
|  | POX-1-R | AGATCGTGTGCTGAGAAACC | 57.9 |
|  | POX-2-F | GCCCAACATTTTGCAGGATC | 57.9 |
|  | POX-2-R | GCTAGACTAGGTTTCGGCAA | 58.3 |
|  | POX-3-F | AACGACGCATGACACAGATT | 58.2 |
|  | POX-3-R | AAGCACTGCATCAAGGTAGG | 57.9 |
|  | POX-4-F | AGAACATTGAGCACCAGAGC | 58.1 |
|  | POX-4-R | TTTATCGCCGTGTTTCTCGT | 57.9 |
| Polyphenol Oxidase | PPO-1-F | GCACAACCTCTGGATCATGT | 57.8 |
|  | PPO-1-R | AGAGCGGTAATAGGTGTCGT | 58.2 |
|  | PPO-2-F | ATGACTCCACGATAAACCGC | 58.1 |
|  | PPO-2-R | ACCGCTGTTGGAAATGTACA | 57.7 |
|  | PPO-3-F | AGAGGTGGAAGTAGCACTGT | 57.9 |
|  | PPO-3-R | TGCTTCATGTCACAGATGGG | 58.8 |
|  | PPO-4-F | GCGCCTACCTGTACTTCTTC | 58.1 |
|  | PPO-4-R | GTCGGTCAAGTTCTTCTCCC | 57.9 |
| Actin | Act-F | AGGGACATCAAGGAGAAGCT | 58.2 |
|  | Act-R | CCGATGAAAGAAGGCTGGAA | 57.8 |

*Serine/threonine kinase (STK, transcript no. DN2506), Lipase class-III (Lip-III, transcript no. DN63949), Heat shock protein 70 (HSP70, transcript no. DN1582), Glucoside hydrolase (GH, transcript no. DN15277), UDP Glucosyltransferase (UGT, transcript no. DN51146), Phenyalanine lyase (PAL, transcript no. DN8260), Sucrose transporter (SUT, transcript no. DN48102), Glutamate dehydrogenase (GDH, transcript no. DN254), Mn-superoxide dismutase (MnSOD, transcript no. DN131007), and Lipoxygenase (LOX, transcript no. DN7868), Lipase gene [Lip-1 (acc. no. OQ184871), Lip-2 (acc. no. MZ590565), Lip-3 (acc. no. MZ590564), and Lip-4 (acc. no. OQ304605), Lipoxygenase [LOX-1 (acc. no. OQ184873), LOX-2 (acc. no. OQ184874), LOX-3 (TRINITY_DN10043_c0_g1_i1) & LOX-4 (TRINITY_DN118950_c0_g1)], Peroxidase [POX-1 (TRINITY_DN109983), POX-2 (TRINITY_DN177283), POX-3 (acc. no. PP171489) & POX-4 (TRINITY_DN115862)] and Polyphenol oxidase [PPO-1 (acc. no. PP765147), PPO-2 (TRINITY_DN183701_c0_g1), PPO-3 (TRINITY_DN167165_c0_g1) & PPO-4 (TRINITY_DN112679_c0_g1).
