# Supplementary material for: When genes turn traitor: de novo transcriptomics uncovers pearl millet’s rancidity machinery
Source: Front Plant Sci. 2025 Nov 17;16:1677082. doi: 10.3389/fpls.2025.1677082 (PMC12666563; doi:10.3389/fpls.2025.1677082)
Supplement: Supplementary file 15 [file Table2.docx]

**Table S2.** Summary of transcriptome assembly of data generated from landraces, hybrid and composite of pearl millet using the Trinity assembler with default option.

| Count of Transcripts |  |
| --- | --- |
| Total trinity genes | 219965 |
| Total Trinity transcripts | 386184 |
| Present GC | 47.98 |
| Contig N50 | 2614 |
| Median contig length | 514 |
| Average contig | 1208.13 |
| Total assembled bases | 154468663 |
