# Supplementary material for: When genes turn traitor: de novo transcriptomics uncovers pearl millet’s rancidity machinery
Source: Front Plant Sci. 2025 Nov 17;16:1677082. doi: 10.3389/fpls.2025.1677082 (PMC12666563; doi:10.3389/fpls.2025.1677082)
Supplement: Supplementary file 16 [file Table3.docx]

**Table S3.** Summary of annotation of generated assembled transcripts using rice database.

| **Annotations** |  |
| --- | --- |
| Number of Trinity sequences | 386184 |
| Number of cds sequences from Rice database | 42355 |
| Number of Trinity sequences got homology based on Tophit | 370201 (95.86%) |
| Number of Trinity sequences got gene description based on Rice db | 3,70,201 (95.86%) |
| Number of Rice cds got homology with Trinity sequences | 34890 |
| Number of Trinity sequences remained Unannotated | 15983 |
